# Pregnancy Diagnosed by the Urine

**Published:** 1897-03

**Authors:** 


### PREGNANCY DIAGNOSED BY THE URINE.

Dr. William E. Parke, in following Dr. William D. Gray, of Richmond, Va., states in the *American Gynecological and Obstetrical Journal*, that he can make a positive diagnosis of pregnancy where it existed, within twenty days after conception, by certain changes in the microscopical appearance of the urinary phosphates.

The normal triple phosphate is stellate and markedly feathery. Soon after conception the feathery parts begin to disintegrate, take on the cystals, approach to normal, and at term are normal.

Dr. Gray's directions for preparing the urine for examination are as follows: Take about one inch and a quarter of the suspected urine in a small test tube and add about one-third as much Tyson's magnesian fluid as there is urine. This will throw down the phosphates in fifteen or twenty minutes and furnish the necessary material for microscopic examination. Tyson's fluid is composed of one part each of the muriate of ammonia, aqua ammonia and sulphate of magnesia, and eight parts of distilled water. Dr. Parke recapitulates as follows:

When conception occurs the triple phosphates in the urine change in form. They lose their feathery appearance, the change beginning at the tip and progressing toward the base. One side only may be affected, or both, leaving only the shaft and perhaps a few fragments adhering. The shaft assumes a beaded or jointed appearance. These changes commence within twenty days after conception, and are most marked in the early months and almost absent in the later months. When the death of the fœtus occurs, the phosphates resume their normal appearance. This observation I have not had the opportunity of confirming.

Conclusions:—1. The change in the urinary phosphates pointed out by Dr. Gray occurs in a very large percentage of pregnant women.

2. This change is not equally pronounced in the urine at the same period of gestation in different women, nor at consecutive examinations of the urine of the same woman.

3. When recognized, it forms a strongly presumptive evidence of pregnancy.

4. This sign is recognizable very early. (Dr. Gray, in a personal letter to me, states that he has made many diagnoses as early as ten days after conception.) It is, therefore, of the greatest value when other signs are of the least value or not present at all.

5. A diagnosis of probable pregnancy can be made without a physical examination, or without exciting the suspicion of the patient.—*Maryland Medical Journal.*

